# Gene expression profiling of calcifications in breast cancer

**DOI:** 10.1038/s41598-017-11331-9

**Published:** 2017-09-12

**Authors:** Sung Ui Shin, Jeonghoon Lee, Ju Han Kim, Won Hwa Kim, Sung Eun Song, Ajung Chu, Hoe Suk Kim, Wonshik Han, Han Suk Ryu, Woo Kyung Moon

**Affiliations:** 1 Department of Radiology, Seoul National University Hospital and Seoul National University College of Medicine, Seoul, Korea; 2 Division of Biomedical Informatics, Seoul National University College of Medicine, Seoul, Korea; 3 Department of Surgery, Seoul National University Hospital, Seoul, Korea; 4 Department of Pathology, Seoul National University Hospital, Seoul, Korea

## Abstract

We investigated the gene expression profiles of calcifications in breast cancer. Gene expression analysis of surgical specimen was performed using Affymetrix GeneChip® Human Gene 2.0 ST arrays in 168 breast cancer patients. The mammographic calcifications were reviewed by three radiologists and classified into three groups according to malignancy probability: breast cancers without suspicious calcifications; breast cancers with low-to-intermediate suspicious calcifications; and breast cancers with highly suspicious calcifications. To identify differentially expressed genes (DEGs) between these three groups, a one-way analysis of variance was performed with post hoc comparisons with Tukey’s honest significant difference test. To explore the biological significance of DEGs, we used DAVID for gene ontology analysis and BioLattice for clustering analysis. A total of 2551 genes showed differential expression among the three groups. ERBB2 genes are up-regulated in breast cancers with highly suspicious calcifications (fold change 2.474, *p* < 0.001). Gene ontology analysis revealed that the immune, defense and inflammatory responses were decreased in breast cancers with highly suspicious calcifications compared to breast cancers without suspicious calcifications (*p* from 10^−23^ to 10^−8^). The clustering analysis also demonstrated that the immune system is associated with mammographic calcifications (*p* < 0.001). Our study showed calcifications in breast cancers are associated with high levels of mRNA expression of ERBB2 and decreased immune system activity.

## Introduction

Mammography is an established screening tool for breast cancer and calcifications are one of the most important findings for the detection of breast cancer^1, 2^. In addition, the detection and characterization of calcifications are important in preoperative evaluations of lesion extent and surveillance after treatment in breast cancer patients^3, 4^. There are well-established diagnostic criteria based on morphology and distribution of the calcifications at radiologic examination^5, 6^. Evidence suggests that calcifications affect the prognosis of breast cancer, such that breast cancers with calcifications are predicted to be more aggressive and have worse prognosis than those without calcifications^7–13^. Tabar *et al*. and others have reported that cancers with calcifications of different morphology have different outcomes^9, 14^.

Previous studies have attempted to evaluate and interpret the relationship between mammographic calcifications and tumor histology or the expression of selected biological markers, such as estrogen receptor (ER), progesterone receptor (PR) and human epidermal growth factor receptor 2 (HER2), using immunohistochemistry and have shown that breast cancers with mammographic calcifications are more frequently associated with invasive cancer with extensive ductal carcinoma *in situ* (DCIS) or HER2-positive breast cancer^15–20^. However, the underlying molecular biology involving calcifications remains poorly understood and needs to be investigated to provide more clues that would help breast specialists make treatment decisions preoperatively^5^.

Linking the relationships between clinical, imaging, pathologic and genomic data is called radiogenomics and there has been growing interest in exploring multiscale relationships in human breast cancers^21–24^. In breast cancer, the mainstay of radiogenomic studies often uses magnetic resonance imaging and showed association between several imaging features and genomic data. Identifying the biological background associated with progression and tumor aggressiveness in conjunction with imaging features, such as calcifications, could yield additional data that could assist in pretreatment planning and discussion of prognosis, as well as add to our understanding of tumor biologic characteristics^18, 19^. However, to our knowledge, the relationships between mammographic imaging findings and global transcriptomic profiles are not reported. The purpose of this study was to investigate the gene expression profiles of calcifications in breast cancer.

## Results

### Patient characteristics

Demographic characteristics and clinicopathologic findings are summarized in Table 1. The median patient age was 50.0 years. The mean tumor size for the study group was 3.1 cm. Patients had invasive ductal carcinomas (89.9%), invasive lobular carcinomas (1.8%) or others with a clinical stage of I (14.3%), II (63.1%), or III (22.6%). Ninety-two patients were hormone receptor (HR) - positive and the other 76 were HR - negative. For HER2 receptor status, 38 patients were positive, and 130 were negative. HER2 positivity, DCIS and comedo necrosis (all *p* < 0.001) were more frequently observed in breast cancers with highly suspicious calcifications. The other demographic, clinical and pathologic findings were not significantly different between the three groups.

**Table 1:** Patient characteristics according to mammographic calcifications.

	Breast cancer without suspicious calcifications (n = 99)	Breast cancer with low-to-intermediate suspicious calcifications (n = 37)	Breast cancer with highly suspicious calcifications (n = 32)	*p* value
**Clinicopathologic variable**				
Age, years				0.383
≤50	53 (53.5)	16 (43.2)	19 (59.4)	
>50	46 (46.5)	21 (56.8)	13 (40.6)	
Menopausal status				0.191
Premenopausal	51 (51.5)	13 (35.1)	18 (56.3)	
Postmenopausal	48 (48.5)	23 (62.2)	14 (43.8)	
Clinical symptom*				0.151
Yes	91 (91.9)	30 (81.1)	27 (84.4)	
No	8 (8.1)	7 (18.9)	5 (15.6)	
Mean tumor size ± SD (cm)^†^	3.2 ± 1.2	3.1 ± 1.3	2.7 ± 2.1	0.179
Lymph node metastasis				0.255
Yes	43 (43.4)	19 (51.4)	10 (31.3)	
No	56 (56.6)	18 (48.7)	22 (68.8)	
Histologic grade				0.799
I-II	31 (31.3)	10 (27.0)	11 (34.4)	
III	68 (68.7)	27 (73.0)	21 (65.6)	
HR positivity	55 (55.6)	22 (59.5)	15 (46.9)	0.560
HER2 positivity	12 (12.1)	10 (27.0)	16 (50.0)	<0.001
Triple-negative	33 (33.3)	11 (29.8)	5 (15.6)	0.159
DCIS	58 (58.6)	28 (75.7)	31 (96.9)	<0.001
Comedo necrosis	23 (23.2)	21 (56.8)	29 (90.6)	<0.001
**Mammographic findings**				
Breast composition				0.122
Fatty	34 (34.3)	10 (27.0)	5 (15.6)	
Dense	65 (65.7)	27 (73.0)	27 (84.4)	
Mammographic finding				<0.001
Mass with calcifications	0 (0)	36 (97.3)	28 (87.5)	
Mass without calcifications	88 (88.9)	0 (0)	0 (0)	
Asymmetry or architectural distortion	11 (11.1)	1 (2.7)	4 (12.5)	
Mass shape*^‡^				0.003
Oval or round	26 (29.5)	12 (33.3)	0 (0)	
Irregular	62 (70.5)	24 (66.7)	28 (100.0)	
Mass margin*^‡^				0.141
Circumscribed	11 (12.5)	4 (11.1)	0 (0)	
Not circumscribed	77 (87.5)	32 (88.9)	28 (100.0)	
Mass density*^‡^				0.132
High	77 (87.5)	30 (83.3)	24 (85.7)	
Equal	11 (12.5)	6 (16.7)	4 (14.3)	

*HR*, hormone receptor. *HER2*, human epidermal growth factor receptor 2. *DCIS*, ductal carcinoma *in situ*. Unless otherwise indicated, the data are the number of patients, and the data in parentheses are percentages. *p value was calculated using Fisher’s exact. Others were calculated using chi-square test. ^†^The numbers represent the mean value ± standard deviation. The *p* value was calculated using ANOVA. ^‡^Shape, margin and density of mass were evaluated only in mass cases.

### Mammographic features analysis

Mammographic features of the patients are shown in Table 1. The majority of cases presented as mass with or without calcifications (*p* < 0.001). There was no significant difference between the three groups for breast composition or margin and the density of mass. The morphology of calcifications was coarse heterogeneous (n = 1), fine pleomorphic (n = 21), and fine linear and linear branching (n = 10), and the distribution was regional (n = 1), grouped (n = 12), and segmental (n = 19).

### Imaging-genomic correlation

When we compared the genomic composition of the three groups, 2551 genes were differentially expressed at the level of *p* < 0.05. Of these, 1838 DEGs (955 up and 883 down) were detected in breast cancers with highly suspicious calcifications compared to those without suspicious calcifications, 484 DEGs (342 up and 142 down) were detected in breast cancers with highly suspicious calcifications compared to those with low-to-intermediate suspicious calcifications, and 457 DEGs (126 up and 331 down) were detected in breast cancers with low-to-intermediate suspicious calcifications compared to those without suspicious calcifications.

The lists of DEGs in all three comparison sets are shown in Supplementary Table 1. With these DEG sets, gene ontology analyses were performed with DAVID. Top 20 genes ordered by *p* value and fold change in breast cancers with highly suspicious calcifications compared to those without suspicious calcifications are shown in Tables 2 and 3, respectively. There were three genes which are repetitively shown on Tables 2 and 3. ERBB2 gene is up-regulated in breast cancers with highly suspicious calcifications compared to those with low-to-intermediate suspicious calcifications or those without suspicious calcifications. There was no difference in expression levels of ERBB2 between breast cancers with low-to-intermediate suspicious calcifications and those without suspicious calcifications. COL11A1 is down-regulated in breast cancers with highly suspicious calcifications compared to those with low-to-intermediate suspicious calcifications (*p* = 0.002) and those without suspicious calcifications (*p* = 1.98E-06). However, there was no difference in expression levels of COL11A1 between breast cancers with low-to-intermediate suspicious calcifications and those without suspicious calcifications (*p* = 0.489). FNDC1 is up-regulated in breast cancers without suspicious calcifications compared to those with highly suspicious calcifications (*p* = 4.67E-06) and low-to-intermediate suspicious calcifications (*p* = 0.014). However, there was no difference between breast cancers with highly suspicious and those with low-to-intermediate suspicious calcifications (*p* = 0.131).

**Table 2:** Top 20 genes ordered by *p* value in breast cancer with highly suspicious calcifications compared to those without suspicious calcifications.

NCBI ID	HUGO	Gene Name	*p* value	Fold change
94103	ORMDL3	ORMDL sphingolipid biosynthesis regulator 3	2.5E-07	1.582
3038	HAS3	hyaluronan synthase 3	5.8E-07	1.304
338557	FFAR4	free fatty acid receptor 4	7.8E-07	0.758
64175	P3H1	prolyl 3-hydroxylase 1	1.4E-06	0.771
1301	COL11A1	collagen, type XI, alpha 1	2.0E-06	0.400
54894	RNF43	ring finger protein 43	2.8E-06	1.603
84624	FNDC1	fibronectin type III domain containing 1	4.7E-06	0.439
6507	SLC1A3	solute carrier family 1 (glial high affinity glutamate transporter), member 3	5.4E-06	0.677
3983	ABLIM1	actin binding LIM protein 1	7.1E-06	1.461
5996	RGS1	regulator of G-protein signaling 1	7.5E-06	0.531
1501	CTNND2	catenin (cadherin-associated protein), delta 2	8.8E-06	1.530
2064	ERBB2	erb-b2 receptor tyrosine kinase 2	9.0E-06	2.474
80896	NPL	N-acetylneuraminate pyruvate lyase (dihydrodipicolinate synthase)	9.5E-06	0.724
5328	PLAU	plasminogen activator, urokinase	9.7E-06	0.583
713	C1QB	complement component 1, q subcomponent, B chain	1.0E-05	0.681
822	CAPG	capping protein (actin filament), gelsolin-like	1.1E-05	0.833
1573	CYP2J2	cytochrome P450, family 2, subfamily J, polypeptide 2	1.3E-05	1.738
586	BCAT1	branched chain amino-acid transaminase 1, cytosolic	1.3E-05	0.673
2706	GJB2	gap junction protein beta 2	1.5E-05	0.511
10082	GPC6	glypican 6	1.7E-05	0.596

**Table 3:** Top 20 genes ordered by fold change in breast cancer with highly suspicious calcifications compared to those without suspicious calcifications.

NCBI ID	HUGO	Gene Name	*p* value	Fold change
118430	MUCL1	mucin-like 1	8.2E-04	12.962
7021	TFAP2B	transcription factor AP-2 beta (activating enhancer binding protein 2 beta)	2.5E-02	5.511
4680	CEACAM6	carcinoembryonic antigen-related cell adhesion molecule 6 (non-specific cross reacting antigen)	3.8E-02	4.977
730	C7	complement component 7	4.8E-04	4.629
219970	GLYATL2	glycine-N-acyltransferase-like 2	6.1E-02	4.263
9635	CLCA2	chloride channel accessory 2	9.4E-05	4.174
339479	BRINP3	bone morphogenetic protein/retinoic acid inducible neural-specific 3	4.0E-04	4.088
79983	POF1B	premature ovarian failure, 1B	8.8E-05	3.583
4322	MMP13	matrix metallopeptidase 13	3.1E-04	0.320
85320	ABCC11	ATP-binding cassette, sub-family C (CFTR/MRP), member 11	2.5E-02	2.776
26154	ABCA12	ATP-binding cassette, sub-family A (ABC1), member 12	8.8E-03	2.666
1300	COL10A1	collagen, type X, alpha 1	2.2E-03	0.381
3081	HGD	homogentisate 1,2-dioxygenase	3.6E-04	2.585
646424	SPINK8	serine peptidase inhibitor, Kazal type 8 (putative)	4.1E-02	2.513
1301	COL11A1	collagen, type XI, alpha 1	2.0E-06	0.400
2064	ERBB2	erb-b2 receptor tyrosine kinase 2	9.0E-06	2.474
10351	ABCA8	ATP-binding cassette, sub-family A (ABC1), member 8	1.5E-03	2.436
90865	IL33	interleukin 33	4.6E-05	2.377
84624	FNDC1	fibronectin type III domain containing 1	4.7E-06	0.439
131578	LRRC15	leucine rich repeat containing 15	1.2E-04	0.443

**Figure 1:**
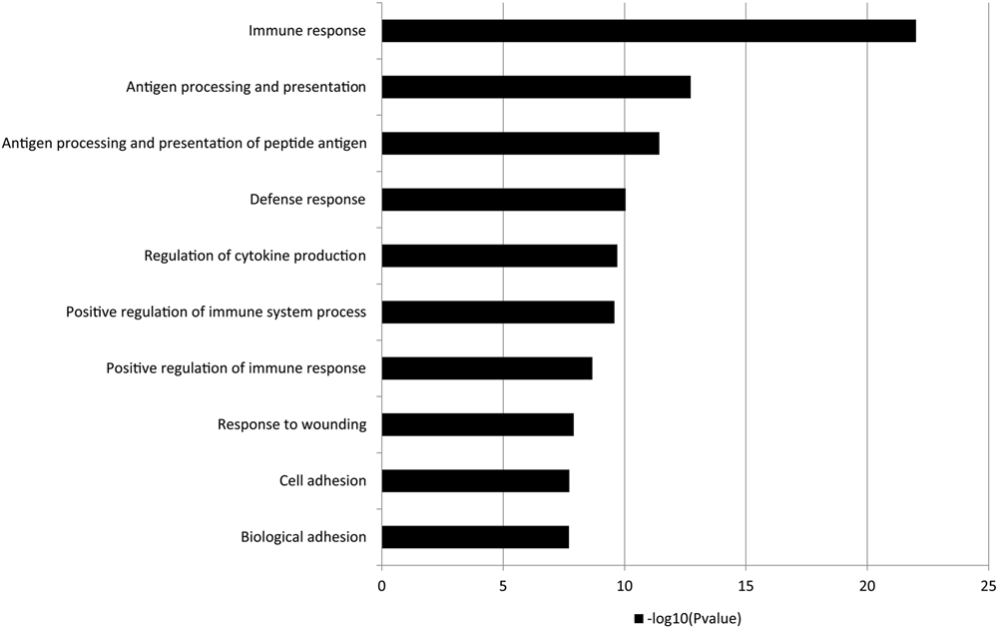
Enriched gene ontology biologic process terms using down-regulated DEGs (n = 882) from the comparison between breast cancers with highly suspicious calcifications and those without suspicious calcifications. Immune system-related GO terms in the biological process category are enriched in breast cancers without suspicious calcifications.

Focusing on down regulated genes, we found that the 10 top-ranked biological functions (*p* from 10^−23^ to 10^−8^, Figure 1) included the immune response, antigen processing and presentation, defense response, the regulation of cytokine production, the positive regulation of immune system and response to wounding. In breast cancers with highly suspicious calcifications compared to those with low-to-intermediate suspicious calcifications, gene ontology biological process terms included two dominant functions; skeletal system development and immune response. Focusing on down regulated genes, we found that top-ranked biological functions (*p* < 0.05) included cartilage development, skeletal system development, limb morphogenesis, osteoblast differentiation, ossifications, immune response, leukocyte mediated immunity, and response to wounding. In breast cancers with low-to-intermediate suspicious calcifications compared to those without suspicious calcifications, focusing on down regulated genes, 10 top-ranked biological functions (*p* from 10^−6^ to 10^−2^) included antigen processing and presentation, regulation of apoptosis, cytokine mediated signaling pathway, regulation of programmed cell death, and defense response.

**Figure 2:**
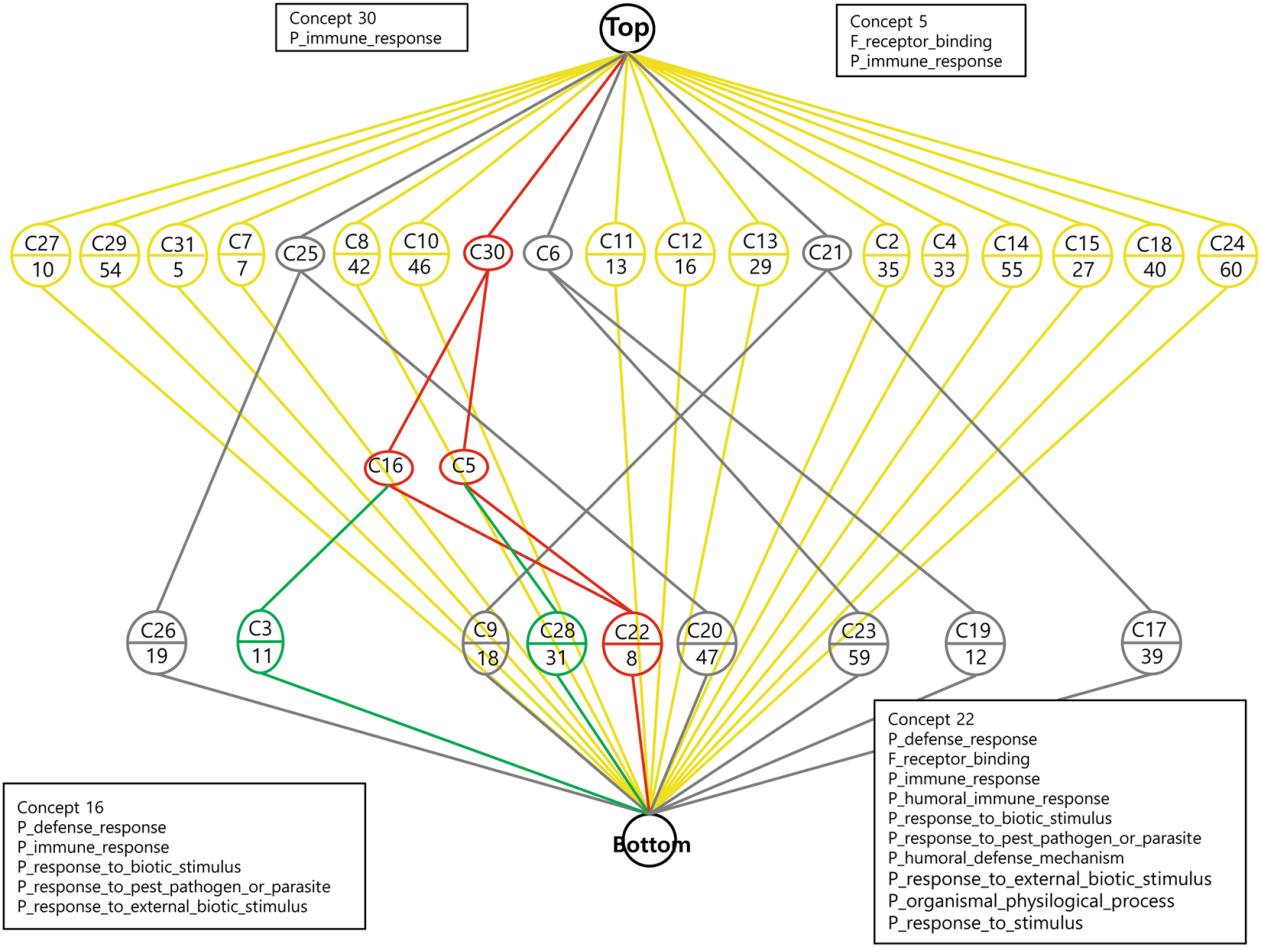
Concept lattice constructed from the comparison between breast cancers with highly suspicious calcifications and those without suspicious calcifications (n = 1838) in a total population with 60 clusters annotated by GO terms in the biological process category. Only 24 of 60 clusters demonstrate at least one significant GO term(s) (*p* < 0.001). Overall, the dataset shows 125 significant annotations with 106 unique GO terms. The core–periphery substructures are marked with colors (i.e., core in red, communicating in green, independent in yellow and peripheral in gray).

**Figure 3:**
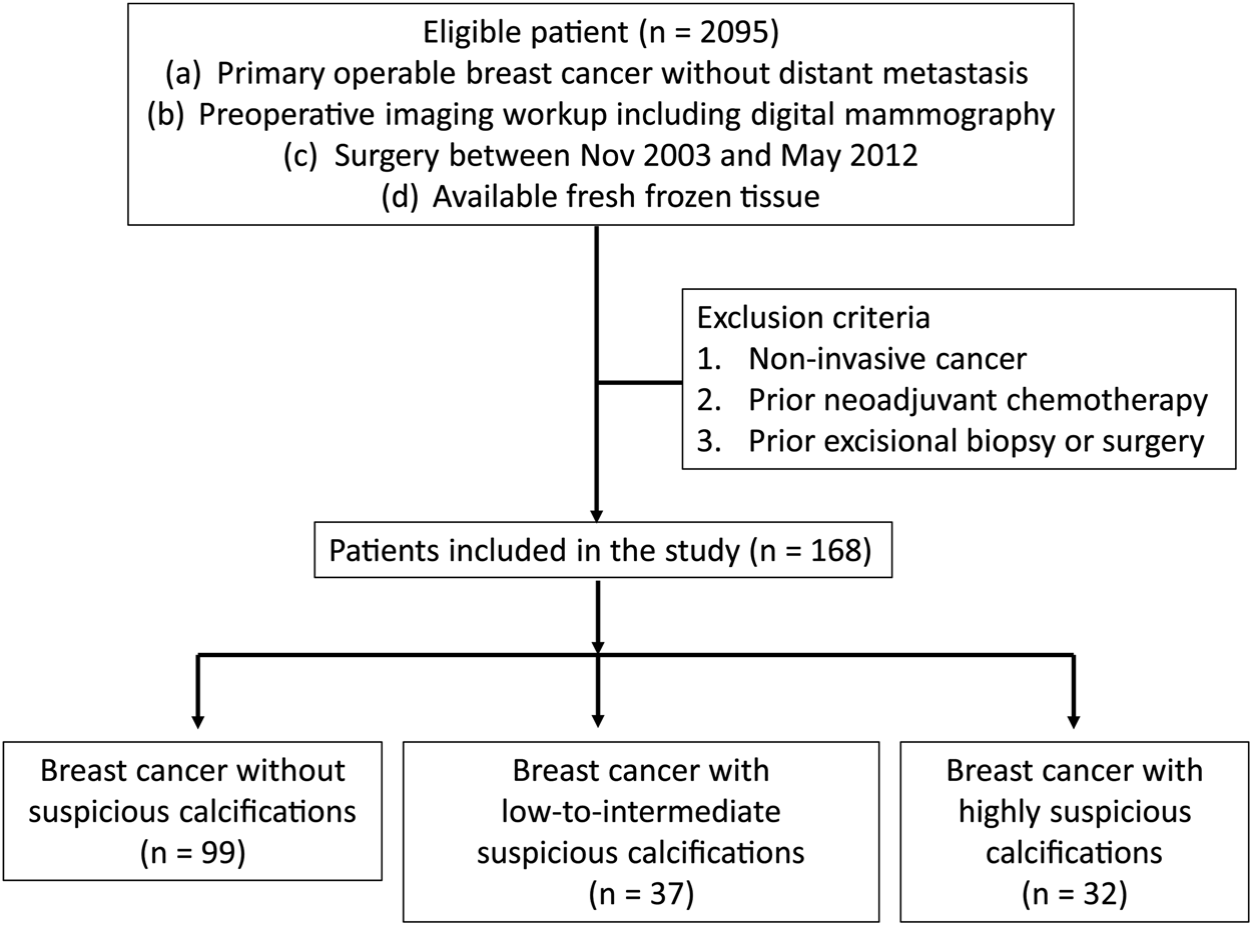
Schematic of the inclusion and exclusion criteria and the selection process of this study cohort.

**Figure 4:**
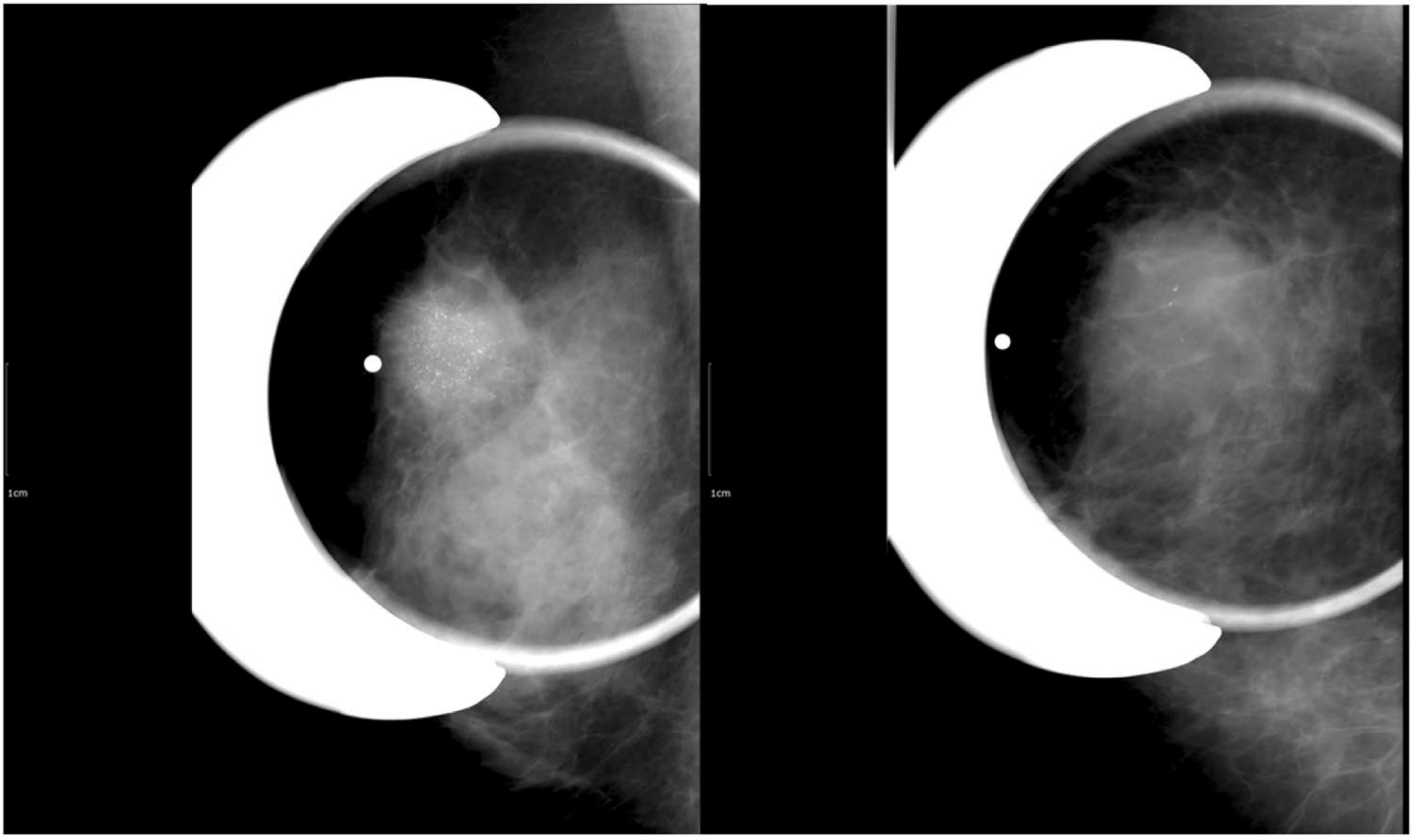
Examples of calcifications. (**A**) A 55-year-old woman presented with palpable mass in the right breast and magnification mammogram revealed not circumscribed hyperdense mass with grouped fine pleomorphic and fine linear calcifications in the right upper breast. She was categorized as highly suspicious calcifications group and diagnosed as 2.8 cm invasive ductal carcinoma with comedo necrosis. Immunohistochemistry analysis revealed that the tumor was ER-negative, PR-negative, and HER2-positive cancer. (**B**) A 50-year-old woman presented with palpable mass in the right breast and magnification mammogram revealed not circumscribed hyperdense mass with grouped coarse and amorphous calcifications in the right upper breast. She was categorized as low-to-intermediate calcifications group and diagnosed as 4.5 cm invasive ductal carcinoma. Immunohistochemistry analysis revealed that the tumor was ER-negative, PR-negative, and HER2-negative cancer.

BioLattice analysis identified the lattice of concepts constructed with DEGs between breast cancers with highly suspicious calcifications and those without suspicious calcifications with 60 clusters annotated by gene ontology (GO) terms in the biological process category (Figure 2). Only 24 of 60 clusters demonstrated at least one significant GO term(s) (*p* < 0.001). Overall, the dataset showed 125 significant annotations with 106 unique GO terms. Four core concepts (shown as red color) are associated with the immune system, including defense response, immune response, and inflammatory response. With DEGs between breast cancers with highly suspicious calcifications and those with low-to-intermediate suspicious calcifications, lattice of concept was constructed with 17 clusters annotated by GO terms in the biological process category. Four clusters had significant GO terms (*p* < 0.001). It also contained defense response and immune response. In the comparison of breast cancers with low-to-intermediate suspicious calcifications and those without suspicious calcifications, lattice of concept was made with 16 clusters annotated by GO terms in the biological process category. Only 1 cluster demonstrated significant GO terms (*p* < 0.001). It was composed of immune response, response to pest pathogen or parasite, and response to external biotic stimulus.

### Analysis of tumor infiltrating lymphocytes

The mean tumor infiltrating lymphocyte (TIL) score was 37.0 ± 29.5 for breast cancers with highly suspicious calcifications, 34.9 ± 30.4 for those with low-to-intermediate suspicious calcifications, and 42.4 ± 32.8 for those without suspicious calcifications. There was no significant difference between three groups (*p* = 0.504) in total 130 patients. Subgroup analysis according to immunohistochemistry results revealed that there was no significant difference between three groups.

When we combine breast cancers with highly suspicious and low-to-intermediate suspicious calcifications into breast cancers with suspicious calcifications (n = 52) and then compare the mean TIL score of it with that of breast cancers without suspicious calcifications (n = 78), there was no significant difference between two groups (35.9 vs. 42.4, *p* = 0.251). However, in triple-negative subtype, the mean TIL score was significantly lower in breast cancers with suspicious calcifications than those without suspicious calcifications (41.5 vs. 62.7, *p* = 0.045). The other subgroups didn’t show the statistical significant differences.

## Discussion

We searched for gene expression profiles of breast cancers with suspicious calcifications and compared those with gene expression profiles of breast cancers without suspicious calcifications. Gene expression patterns were different according to the status of mammographic calcifications in breast cancer. First, breast cancers with highly suspicious calcifications are associated with high levels of mRNA expression of ERBB2 and decreased expression of COL11A1 and FNDC1. Second, GO and clustering analysis using DAVID and BioLattice revealed that breast cancer patients with highly suspicious calcifications on mammography were highly associated with decreased immune system activity.

In our experiments, ERBB2 is repetitively overexpressed shown on the list of top 20 genes ordered by *p* value or fold change and commercially available gene signatures in PAM50, MammaPrint® and OncotypeDX® (Tables 2–4). To best of our knowledge, it is the first report that insists relationship between mammographic calcifications and mRNA expression of ERBB2. In addition, it supports the findings of other studies and provides bridging evidence that showed there was an association between HER2 overexpression and calcifications in breast cancer patients^16, 18, 19, 25, 26^. Yepes *et al*.^27^ reported that a mass with pleomorphic calcifications on mammography may predict an intermediate to high recurrence score in patients with stage I-II ER-positive, HER2-negative, and lymph node negative invasive breast cancer. Similarly, Chae *et al*.^28^ also reported that the high risk group assessed by 21-gene recurrence score assays was associated with the presence of calcification in the mass and the absence of calcification in the mass is independent predictors associated with low recurrence score. In our study, breast cancers with suspicious calcifications had low expression of COL11A1 and FNDC1. There is little known about FNDC1 gene. COL11A1 is an extracellular matrix molecule which plays an important role in endochondral ossification^29^. In addition, there are evidences that COL11A1 overexpression is related with up- regulation of TGF-β1 and a biomarker indicating activated cancer associated fibroblasts in several epithelial cell origin cancers^30, 31^. Several articles reported that co-cultures of cancer associated fibroblasts with breast cancer cells increased metastatic ability^32, 33^. It is known to be associated with tumor aggressiveness, tumor progression, infiltration, metastasis and poor survival in several cancers^30, 34, 35^. However, Fuentes-Martínez *et al*.^36^ reported that COL11A1 is a stromal marker but does not have prognostic value in breast cancer. To sum up these findings, breast cancer with suspicious calcifications would have another pathway for calcifications formation rather than endochondral ossification and would have little association with stromal remodeling.

**Table 4:** Comparison of commercially available gene signatures in PAM50, MammaPrint® and OncotypeDX®.

NCBI ID	Gene symbol	*p* value	Fold change		
			Breast cancer with highly suspicious calcifications compared to those without suspicious calcifications	Breast cancer with highly suspicious calcifications compared to those with low-to-intermediate suspicious calcifications	Breast cancer with low-to-intermediate suspicious calcifications compared to those without suspicious calcifications
2064	ERBB2	1.6E-05	2.474	2.254	1.098
2886	GRB7	6.3E-05	2.122	1.875	1.132
968	CD68	1.5E-04	0.727	0.841	0.864
8030	CCDC6	9.2E-04	1.131	1.055	1.072
120224	TMEM45B	9.5E-04	1.859	1.589	1.170
8840	WISP1	2.3E-03	0.798	1.011	0.789
4318	MMP9	3.5E-03	0.728	1.092	0.666
4320	MMP11	5.0E-03	0.682	0.771	0.885
2321	FLT1	7.2E-03	1.183	1.179	1.003
58475	MS4A7	1.0E-02	0.711	0.680	1.045
26996	GPR160	1.0E-02	1.197	1.106	1.082
643008	SMIM5	1.9E-02	1.079	1.057	1.020
3861	KRT14	4.0E-02	1.898	1.623	1.169
2264	FGFR4	4.1E-02	1.298	1.194	1.087
3169	FOXA1	4.8E-02	1.008	0.945	1.067

To the best of knowledge, it is the first report that breast cancers with mammographic calcifications are associated with decreased immune system activity. Although the direct cellular mechanisms or biologic pathways between calcifications and the immune system have not yet been discovered, we could find one possible explanation. Tse *et al*.^37^ reported that rapidly proliferating tumor cells that consume the bloody supply result in tumor necrosis and subsequent acidosis in the microenvironment, which finally causes calcium accumulation in the ducts. We can assume that activated immune system may deter the proliferation of tumor cells and necrosis caused by hypoxia^38, 39^. By contrast, breast cancers with decreased antitumor immune response would have uncontrolled tumor cell proliferation and tumor necrosis, finally causing calcifications in the ducts. Therefore, it is possible that breast cancers with suspicious calcifications are associated with decreased immune system activity. However, there is a conflict within our results showing that breast cancers with suspicious calcifications are associated with rapidly proliferating tumor cells following decreased immune system activity. Because DCIS is an indolent non-invasive tumor but it is frequently associated with mammographic calcifications^40, 41^. The mechanisms by which suspicious calcifications are produced may vary between invasive cancer and DCIS and it must be evaluated in future studies. The mean TIL score was significantly lower in breast cancers with suspicious calcifications than breast cancers without suspicious calcifications only in triple-negative subtype. The results of TIL scores partly supports our gene expression analysis that breast cancers with suspicious calcifications are associated with decreased immune system activity. It might suggest that the associations between calcifications and immune system are strong and apparent in triple-negative subtype than others.

Our study has several limitations. First, this study has a retrospective design, and there may be selection bias in our database. Second, we do not have independent validation set to support our results. At the time of our study, the Cancer Imaging Archive of breast TCGA data had only 4 patients with a preoperative mammography. In addition, we couldn’t perform analyses regarding survival or recurrence due to lack of long term follow-up data. Thus, we tried to use commercially available gene signatures, however, not all of genes were available on the Affymetrix GeneChip® Human Gene 2.0 ST arrays. To overcome the weakness, we analyzed pathologic TIL scores of the same population. However, there remains limitation that we simply hypothesized that TIL might be an indicator of immune system activity, even though immune system is very complex and interactive system. Third, the interpretation of mammographic calcifications is subjective, and other imaging findings were not considered in this study. Fourth, breast cancer is a heterogeneous disease regarding its gene expression profiles. It is possible that the differences between the groups are not only due to the calcification status but also to the intrinsic subtype of breast cancer. Finally, we did not perform *in vitro* experiments regarding the cellular mechanism of calcium deposit or *ex vivo* experiments to determine whether immune cells differentially exist or whether immune cell markers are differentially presented according to mammographic calcifications. Similarly, we did not perform analysis of specific mineral species in the specimen. However, our study has several distinct strengths. Having both fresh frozen tissues obtained from surgical specimen and initial digital mammography is a rare and valuable resource. Additionally, to the best of our knowledge, this is the first study examining the global gene expression profiles of calcifications in breast cancer, and it is the largest study in which over one hundred patients with microarray data were enrolled and that correlates microarray data and imaging features of breast cancer. In addition, our results may guide further studies in the study of biological process or cell signaling pathways of calcification formation.

In conclusion, gene expression patterns in breast cancer are different according to mammographic calcifications. Breast cancers with highly suspicious calcifications are associated with high levels of mRNA expression of ERBB2 and decreased immune system activity. These results, if validated, could be used as the basis for future hypothesis-based studies.

## Methods

### Study population

The institutional review board of Seoul National University Hospital (IRB No. 1409-128-612) approved this retrospective study, and all patients provided written informed consent for their breast cancer tissue to be used for genome sequencing (IRB No. 1405-088-580) before operation. All experiments were performed in accordance with relevant guidelines and regulations. We retrospectively identified 2095 consecutive patients with primary operable breast cancer who performed preoperative imaging workup, and underwent surgery between 2003 and 2012 from the Breast Imaging Center database of Seoul National University Hospital. We excluded patients who had (a) non-invasive cancer, (b) prior neoadjuvant chemotherapy; or (c) prior excisional biopsy or breast surgery. As a result, a total of 168 women (mean, 50.7 yrs; age range, 21–79 yrs) comprised our study group (Figure 3).

### Mammography acquisition and analysis

Mammography was performed using a Senograph 2000D or Senograph DS (GE Healthcare, Milwaukee, WI, USA) or a LORAD Selenia (Hologic, Boston, MA, USA) digital mammography unit. Standard two-view mammography was performed with additional views as necessary. A Senograph system was used on 104 (61.9%) women, and a Selenia system was used on 64 (38.1%) women.

Mammographic features of the patients were assessed according the Breast Imaging-Reporting and Data System (BI-RADS)^6^. Using calcifications at mammography as a criterion for grouping the patients, three radiologists (S.E.S., A.C., and W.K.M.) with different degrees of experience in interpreting mammography independently analyzed the calcifications without access to genomic data. The radiologists had to fill out a sheet for each case giving their BI-RADS category: 1, normal; 2, benign; 3, probably benign; 4A, low suspicious; 4B, intermediate suspicious; 4C, highly suspicious; and 5, highly suggestive of cancer. After each radiologist finished the analysis, final consensus was established for each case. Cases with BI-RADS category of 4C and 5 were classified as breast cancer with highly suspicious calcifications (n = 32), cases with BI-RADS category of 3, 4 A, and 4B as breast cancer with low-to-intermediate suspicious calcifications (n = 37), and the other cases with BI-RADS category 1 or 2 as breast cancer without suspicious calcifications (n = 99). For example, fine pleomorphic, fine linear or fine linear branching calcifications were classified as highly suspicious calcifications whereas amorphous or coarse heterogeneous calcifications were classified as low-to-intermediate suspicious calcifications^6, 7^﻿ (Figure 4).

### Tissue samples and Microarray analysis: RNA Isolation, Preparation, Hybridization, and Data Acquisition

Tissue samples were dissected through the centers of the carcinomatous region during surgery at our hospital between 2003 and 2012. These samples were frozen in liquid nitrogen within 20 min following surgical devascularization and stored at −80 °C. Total RNA from each sample was extracted using TRIzol^®^ reagent (Invitrogen, Carlsbad, CA, USA). RNA quality was assessed with an Agilent 2100 Bioanalyzer using the RNA 6000 Nano Chip (Agilent Technologies, Amstelveen, The Netherlands), and the quantity was determined with an ND-2000 spectrophotometer (Thermo Inc., DE, USA). The median RNA extracted was 1.202 g/L (range, 0.143–2.986 g/L). Total RNA was measured as the UV absorbance at 260 nm. Sample purity was assessed by measuring the OD 260:280 nm and OD 260:230 nm. The integrity of RNA samples was confirmed by the appearance of distinct 28S and18S bands of ribosomal RNA. The RNA integrity number (RIN) was determined using the RIN algorithm of the Agilent 2100 Expert Software^42^. The quality of the RNA was good with a standard 260/280 ratio and 260/230 ratio of absorbance greater than 1.7 and 1.3 per sample, respectively. The mean 28S/18S ratio was 1.0 (range, 0.3–1.9), and the RIN was greater than or equal to 5.0.

The sample preparation was performed according to the instructions and recommendations provided by the manufacturer. Per RNA sample, 300ng was used as input into the Affymetrix procedures as recommended by protocol (http://www.affymetrix.com/). RNA samples were converted to double-strand cDNA. Using a random hexamer incorporating a T7 promoter, amplified RNA (aRNA) was generated from the double-strand cDNA template though an *in vitro* transcription reaction and purified with the Affymetrix sample cleanup module. The cDNA was regenerated through a random-primed reverse transcription using dNTP mix containing dUTP. The cDNA was then fragmented by UDG and APE 1 restriction endonucleases and end-labeled by terminal transferase reaction incorporating a biotinylated dideoxynucleotide. Fragmented and end-labeled cDNAs were hybridized using the GeneChip Human Gene 2.0 ST oligonucleotide arrays (53,617 probes) for 16 hours at 45 °C and 60 rpm, as described in the Gene Chip Whole Transcript (WT) Sense Target Labeling Assay Manual (Affymetrix). After hybridization, chips were stained and washed in the Genechip Fluidics Station 450 (Affymetrix). An Affymetrix Model 3000 G7 scanner and Affymetrix Command Console Software 1.1 were used for scanning and data extraction. The raw CEL file containing intensity data was used for further analysis. For normalization, the robust multiarray-average algorithm was used^43^, which was developed in the Speed Lab at UC Berkeley.

### Statistical analysis and bioinformatics analysis

Demographic characteristics, clinical, pathologic, and mammographic findings were compared between groups using the chi-square test and Fisher’s exact test for categorical variables. A one-way analysis of variance (ANOVA) was performed for numerical variables.

Statistical analyses of microarray data were performed using R software, version 3.2.4 (http://www.r‐project.org/). The R oligo package was used for processing microarray data, which is freely available on the internet (http://www.bioconductor.org/)^44, 45^. To identify differentially expressed genes between the three groups, a one-way ANOVA was performed with post hoc comparisons with Tukey’s honest significant difference test^46^, and a *p* value < 0.05 was applied as the threshold for statistical significance in the subsequent data analysis.

To gain insight into the underlying biology of DEGs related to mammographic calcifications, functional categories enriched in the differentially expressed genes were identified using the functional annotation and clustering tool of the Database for Annotation, Visualization, and Integrated Discovery (DAVID) v6.7 (https://david.ncifcrf.gov/)^47, 48^. The probability that a GO biological process term was overrepresented was determined using a modified Fisher’s exact test, comparing the proportion of genes in the entire genome that are part of that GO term to the proportion of differentially expressed genes that are part of the same GO term^49^.

In addition, to interpret and organize observed biological changes, we used BioLattice (http://www.snubi.org/software/biolattice/), a mathematical framework based on a concept lattice analysis to make associations of gene expression clusters with biological ontologies or biological pathways. BioLattice considers gene expression clusters as objects and annotations as attributes and provides a graphical summary of the order of relationships by arranging them on a concept lattice in an order based on the set inclusion relationship. Rather than interpreting one cluster at a time, BioLattice integrates all gene expression clusters and annotations into a unified framework as a lattice of concepts^50^. We used Pearson correlation as a similarity measure and set arbitrary k as 60, 17 and 16 for the comparisons between breast cancers with highly suspicious calcifications and those without suspicious calcifications, breast cancers with highly suspicious calcifications and those with low-to-intermediate suspicious calcifications, and breast cancers with low-to-intermediate suspicious calcifications and those without suspicious calcifications, respectively to a cluster containing genes from 15 to 30^51^. We selected a threshold of *p* < 0.001. In addition, we compared our results with commercially available gene signatures used to provide recurrence score: PAM50 assay (NanoString Technologies Inc., Seattle, WA, USA)^52, 53^, MammaPrint® 70-gene Breast Cancer Recurrence Assay (Agendia, Huntington Beach, CA, USA)^54, 55^ and The 21-gene Recurrence Score® assay (Oncotype DX®, Genomic Health, Inc., Redwood City, CA, USA)^56, 57^.

### Pathologic validation

Hematoxylin and eosin–stained slides of frozen human tumor tissue were examined per standard protocols for the pathologic diagnosis. Immunohistochemical analysis was performed on formalin-fixed, paraffin-embedded 4-mm tissue sections using primary mouse monoclonal antibodies for ER, PR, and HER2. For equivocal HER2 results (2+), the status was determined using fluorescence *in situ* hybridization^58^.

After gene ontology and BioLattice analyses, we found that breast cancers with suspicious calcifications are associated with decreased immune system activity. Thus, we additionally planned pathologic review regarding TIL as a part of validation. One pathologist (H.S.R., with 10 years of experience) retrospectively reviewed the H&E slides of the patients and assessed TIL score using methodological recommendation of International TILs Working Group 2014^59^. Of total 168 patients, pathologic slides were available for review only in 130 patients (77.4%); 78 patients (78.8%) of breast cancers without suspicious calcifications, 26 patients (70.3%) of breast cancers with low-to-intermediate suspicious calcifications, and 26 patients (81.3%) of breast cancers with highly suspicious calcifications. The mean TIL score was compared between three groups using ANOVA and independent sample t-test.

## Electronic supplementary material

Supplementary table
